# Cattle Farming Activity Monitoring Using Advanced Deep Learning Approach

**DOI:** 10.3390/s26030785

**Published:** 2026-01-24

**Authors:** Muhammad Asim, Bareera Anam, Muhammad Nadeem Ali, Byung-Seo Kim

**Affiliations:** Department of Software & Communications Engineering, Hongik University, Sejong City 30016, Republic of Korea; masim@mail.hongik.ac.kr (M.A.); anambareera@mail.hongik.ac.kr (B.A.); nadeem@mail.hongik.ac.kr (M.N.A.)

**Keywords:** deep learning, dairy cow, animal welfare, activity monitoring, video, camera

## Abstract

Technological advancements have significantly improved cattle farming, particularly in sensor-based activity monitoring for health management, estrus detection, and overall herd supervision. However, such a sensor-based monitoring framework often illustrates several issues, such as high cost, animal discomfort, and susceptibility to false measurement. This study introduces a vision-based cattle activity monitoring approach deployed in a commercial Nestlé dairy farm, specifically one that is estrus-focused, where overhead cameras capture unconstrained herd behavior under variable lighting, occlusions, and crowding. A custom dataset of 2956 Images are collected and then annotated into four fine-grained behaviors—standing, lying, grazing, and estrus—enabling detailed analysis beyond coarse activity categories commonly used in prior livestock monitoring studies. Furthermore, computer vision-based deep learning algorithms are deployed on this dataset to classify the aforementioned classes. A comparative analysis of YOLOv8 and YOLOv9 is provided, which clearly illustrates that YOLOv8-L achieved a mAP of 91.11%, whereas YOLOv9-E achieved a mAP of 90.23%.

## 1. Introduction

Cattle farming remains one of the essential practices in agriculture, as it provides dairy and meat production for human consumption [1]. In the last few years, this industry has experienced significant changes as a result of the constant development of technology, especially in activity tracking. Besides improving animal welfare, this technological integration also increases the efficiency of farms by offering valuable information on cattle behavior [2]. These technologies support health monitoring, identification of estrus behavior, and overall herd management [3].

Traditionally, the surveillance of cattle behavior has been performed by observation, a process that is subjective and labor-intensive [4]. Consequently, demand for automated, accurate, and reliable monitoring methods has increased. Computer vision, machine learning, and sensor technologies have enabled an automated system that can monitor and analyze the behaviors of cattle in real time [5]. These systems use cameras, sensors, and advanced algorithms to record, analyze, and interpret behavioral information in real time [6]. Despite these recent developments, accurately identifying fine-grained cattle behaviors, specifically estrus-related activities, in unconstrained cattle herd environments remains a significant open research challenge. Many existing monitoring solutions depend on wearable sensors, RFID technologies, accelerometer-based devices, or GPS frameworks, which face practical limitations such as substantial deployment costs, ongoing maintenance demands, animal discomfort, and indirect estimation of behavioral states. Moreover, many computer vision-based approaches have been evaluated primarily in controlled or semi-controlled environments and tend to emphasize coarse behavioral categories, rather than estrus-related behaviors that are critical for reproductive management. In contrast, this work focuses specifically on estrus-oriented, fine-grained behavior classification from overhead cameras in a commercial dairy farm and quantifies how different YOLOv8 and YOLOv9 variants perform under frequent occlusions, variable illumination, dynamic weather conditions, and dense, interactive herd structures. YOLO models are a class of deep learning architectures that have been applied to livestock behavior analysis. In practice, these models can improve detection accuracy while reducing processing time in cattle behavior monitoring. These models detect cattle behaviors such as standing, lying, grazing, and estrus using real-time object detection and classification. These models reduce the reliance on manual observation and enable proactive management decisions that are essential for controlling farm health and herd operations [7]. The integration of advanced data analysis with deep learning algorithms helps mitigate challenges such as occlusion and environmental variability, enhancing the robustness of behavior detection under diverse farming conditions. These models are further refined to improve architectural robustness and algorithmic adaptability to real agricultural environments. However, the recent YOLO architectures have demonstrated strong performance on generic object detection benchmarks; their suitability for fine-grained cattle behavior recognition, specifically for the estrus-related activities under realistic farm conditions, remains insufficiently characterized. Moreover, it remains unclear whether architectural advancements in newer models (e.g., YOLOv9) consistently translate into superior performance over more mature versions (e.g., YOLOv8) when applied to limited and highly variable precision livestock datasets.

Current challenges in livestock management include individual animal identification and tracking, early disease detection, large herd sizes, and the labor-intensive process of labeling and monitoring individual animals, which is necessary for their welfare and farm management. Existing solutions, such as RFID-based animal identification [8], wearable sensors [9], and GPS-based animal tracking frameworks [10], still face limitations in accuracy and dependency on the specific environmental conditions (e.g., lighting conditions, weather conditions, and animal movement). This study addresses this gap by examining the feasibility of cameras as a vision-based framework for fine-grained cattle behavior monitoring under diverse farm conditions. We posit that, for this task, increased architectural complexity is insufficient to improve performance, and that carefully optimized YOLOv8 variants may achieve performance comparable to or exceeding that of newer YOLOv9 models, given the dataset scale and behavioral ambiguity. This study differs in its focus on estrus-oriented, fine-grained behavior recognition from overhead cameras in a Nestlé dairy farm, as well as in its systematic analysis of how the variants YOLOv8 and YOLOv9 perform on this task, thereby informing the practical model selection for the management of precision livestock reproduction. The main objective of this study is to provide a substantial comparison of the existing YOLO version with the recent version, specifically for the estrus-specific cattle behavior recognition under authentic commercial farm conditions. The primary focus of this study is to provide a robust object detection algorithm selection, particularly for cattle-related behavior in a bounded farm. Such deployment-focused assessment remains insufficiently examined within precision livestock farming, despite its central importance for practical, large-scale implementation. The main contributions of this study are summarized as follows:

- A curated, vision-based cattle behavior dataset acquired at a commercial Nestlé dairy farm, comprising 2956 images annotated with four behavior categories (standing: 950 images, lying: 800 images, grazing: 800 images, estrus: 406 images). All images were recorded using fixed overhead cameras installed at approximately 8 ft, under diverse lighting and weather conditions.
- A rigorously controlled, application-oriented assessment of eight YOLOv8 and YOLOv9 variants is conducted under uniform training, augmentation, and evaluation protocols, thereby enabling an equitable comparison between architectural maturity and incremental complexity for estrus-specific cattle behavior recognition.
- A quantitative assessment of the accuracy–latency trade-off for each YOLO variant, reporting mAP, precision, and recall, and average per-frame inference time (50–69 ms, depending on model size), to identify configurations appropriate for real-time use in commercial farm settings.
- A focused analysis of estrus recognition demonstrating that estrus constitutes the most challenging behavior category, with lower detection rates than standing and grazing, and examining how occlusion, subtle postural variations, and class imbalance (406 estrus images vs. >800 images in other classes) contribute to this performance disparity.

**Paper Organization:**

Section 1 introduces the study. Section 2 provides a review of the relevant literature. Section 3 outlines the design and methodology. Section 4 covers model training and evaluation. Section 5 discusses the results. Finally, Section 8 provides the conclusion and future research directions provided in Section 9.

## 2. Literature Review

This section surveys related literature in four domains that are directly aligned with the proposed task and dataset: (I) vision-based approaches for monitoring cattle behavior and health, (II) YOLO-based frameworks for livestock detection and behavior recognition, (III) precision livestock farming datasets and deployment-oriented studies, and (IV) temporal and multimodal fusion methodologies for animal behavior analysis.

### 2.1. Cattle Vision-Based Behavior Monitoring

Deep learning has been widely used to model precision livestock farming, primarily due to the requirement of automated, precise, and real-time monitoring of animals in complex farm settings. Computer vision-based algorithms that involve the utilization of the YOLO series of object detection architectures have emerged as a comprehensive component of livestock activity monitoring since they both exhibit high-precision in their object detection capabilities and low-latency in their inference speeds. In the case of real-time identification of cow behavior, a work introduced the YOLOv3 object detection model. The authors acquired image and video data employing several cameras set up at a cow farm [11]. They introduced an additional training layer based on feature extraction and used it with a new activation function called mish that has a smoother curve than the ReLU. The combination of these characteristics ensured the highest indicators of performance, and the work of the model reached 97.0% recall, 98.5% precision, and 97.2% accuracy. Similarly, another study utilized unmanned aerial vehicles (UAVs) to record video footage of cows in open environment [12]. The collected videos were segmented into clips of 4000 frames and divided into training and testing sets using an 80:20 split. The proposed Faster R-CNN model achieved a detection accuracy of 90.13%, indicating the potential of UAV-based monitoring combined with advanced neural networks for cattle observation. Similarly, another study used a Faster R-CNN model to count dairy goats from surveillance videos [13]. To address challenges associated with static backgrounds, the authors applied foreground segmentation to reduce background influence and improve the detection of goats located near frame boundaries. Background subtraction was employed to separate moving objects from the background, while pooling operations were used for dimensionality reduction. Based on the proposed model, an accuracy of 92% was achieved, exceeding 57% and outperforming the standard Faster R-CNN. However, this study used a small, largely unlabeled dataset and recommends applying transfer learning in future work. YOLO-based detectors have been effectively used to solve body detection, localization, and biometric estimation tasks in cattle-centered research. Body parameter determination and real-time body weight estimation algorithm on YOLOv8 have minimized the labor requirement in large-scale farms without compromising accuracy [14]. Cattle and sheep detection YOLOv8 variants have also been optimized to deliver better stability and accuracy with complex backgrounds and environmental conditions in ecological and open-field farming settings [15]. Livestock management systems that run deep learning with YOLOv5 have also shown consistent cattle counting and tracking capabilities, which are also better than standard region-based convolutional networks [16]. Research on indoor cow localization highlights that the effectiveness of model generalization heavily relies on the camera position, perspective, and task-based fine-tuning mechanisms, which are of paramount importance to functionality implementation [17]. Several studies employed the TensorFlow Object Detection API to train SSD- and Faster RCNN–based models using Inception v2 as the feature extractor [18,19,20]. Both these models were trained to predict the cow object bounding boxes on image frames and were designed with detection confidence. However, despite addressing several challenges, the model exhibited limited performance in detecting objects with varying colors, which remains a key limitation.

### 2.2. YOLO-Based Livestock Detection and Behavior

Comparative analyses of YOLOv8 against YOLOv11 in detecting dead chickens reveal that more advanced forms of the models possess greater resilience to being under occlusion, crowding, and varying illumination, like in commercial farms for small objects [21]. To address the scarcity of labeled data, recent studies have explored synthetic data generation in conjunction with YOLOv9, demonstrating that synthetic datasets can improve model generalization while reducing reliance on costly manual annotations [22]. These results emphasize the significance of both architectural and data-centric solutions in deep learning-based livestock systems. Table 1 summarizes the recent development in livestock and agricultural monitoring using deep learning-based methods, highlighting the different aspects, such as sensing setups, learning strategies, health and welfare monitoring, and objective detection, addressed in the literature. The majority of works are based on custom or hybrid datasets and train YOLO-based models because of their high real-time functionality and detection accuracy for various tasks. The table represents the evident movement towards newer versions of YOLO, such as YOLOv8 and YOLOv9, which consistently outperform earlier models in terms of mAP and resilience in unstructured farm environments. Overall, a comparison with existing studies shows that the proposed approach is demonstrating its competitiveness and relevance for real-time cattle activity monitoring using advanced YOLO architectures.

In addition to poultry-specific applications, YOLO-based methods have been applied to general agricultural and livestock monitoring. The use of multi-dataset identification frameworks, which rely on YOLOv9, has shown enhanced detection capability and, at the same time, limited their computational needs and model dimension. Thus, they are convenient for real-time applications in agricultural fields [23]. Specialized livestock-related tasks (detection of feces on pig skin to improve biosecurity and decrease human–animal contact) and the ability of YOLO-based solutions make them suitable for the reconfigured fine-grained monitoring tasks were also demonstrated [27]. Swine farming has offered behavior or recognition to give useful information in monitoring cattle activities. Integration of YOLO and optimized feature-combining networks has been observed to have better behavior recognition of sows and piglets, which results in animal welfare and effective use of farms [25]. Beyond the YOLOv8 and YOLOv9 variants evaluated in this study, recent surveys and benchmarks document a rapidly advancing landscape of object detectors for agricultural settings. Comprehensive reviews of YOLOv1–YOLOv10 in agriculture indicate that the more recent generations (v8–v10) often yield higher mAP and improved computational efficiency for crop, pest, and livestock detection, while simultaneously underscoring that these gains are strongly conditioned on dataset scale and scene complexity. Subsequent architectures such as YOLOv11 and YOLOv12 incorporate enhanced attention mechanisms and refined backbone designs to further balance inference speed and detection accuracy, whereas transformer-based models, including RF-DETR and advanced RT-DETR variants, have reported strong performance on occluded, multi-scale targets in orchards and free-range poultry environments. In contrast to these developments, the present work intentionally restricts its analysis to YOLOv8 and YOLOv9 under a unified evaluation protocol, in order to disentangle the effects of architectural maturity and added complexity on a small, fine-grained, estrus-oriented cattle dataset. Systematic benchmarking against YOLOv10+, YOLOv12 [29], and RF-DETR [30] is therefore identified as a critical direction for future work to more precisely delineate the state-of-the-art for this application.

### 2.3. Precision Livestock Datasets and Deployment

Comprehensive surveys of deep learning-based computer vision systems for livestock applications report their utility in phenotype prediction, behavior analysis, and decision-making, while identifying ongoing challenges such as dataset heterogeneity, limited cross-farm generalization, and deployment constraints [5]. YOLO-based detectors have been effectively used to solve body detection, localization, and biometric estimation tasks in cattle-centered research. Body parameter determining and real-time body weight estimation algorithm on YOLOv8 has minimized the labor requirement in large-scale farms without compromising accuracy [14]. Cattle and sheep detection YOLOv8 variants have also been optimized to deliver better stability and accuracy with complex backgrounds and environmental conditions in ecological and open-field farming settings [15]. Livestock management systems that run deep learning with YOLOv5 have also shown consistent cattle counting and tracking capabilities, and better than standard region-based convolutional networks [16]. Research on indoor cow localization highlights that the effectiveness of model generalization heavily relies on the camera position, perspective, and task-based fine-tuning mechanisms, which are of paramount importance to the functionality implementation [17]. Although contemporary studies focus on vision-based approaches, alternative modalities of sensing have also been investigated. Ruminant behavior prediction methods built upon accelerometers can predict substantial activities reliably on raw sensor measurements but frequently suffer from uncommon or transitional behaviors, which motivates the incorporation of vision-based deep learning to attain more high-resolution and reliable activity tracks [31]. Similar studies on personal animal recognition include sheep facial recognition through multi-dimensional feature fusion, which also provides an example of how deep learning can be used to aid individual-level monitoring and health assessment of complex livestock settings [28].

### 2.4. Temporal and Multimodal Fusion for Behavior Recognition

More recently, real-time detectors (transformer-based) like RT-DETR have been integrated into livestock tracking systems and have been demonstrated to be more effective than traditional YOLO models in identifying cattle maintenance and abnormal behaviors, reflecting an increase in the popularity of hybrid and attention-based detectors for complex activity analysis [24]. Swine farming has offered behavior or recognition to give useful information in monitoring cattle activities. Integration of YOLO and optimized feature-combining networks has been observed to have better behavior recognition of sows and piglets, which results in animal welfare and effective use of farms [25]. In a similar context, the use of spatial–temporal feature fusion in refined YOLOv5 models has demonstrated that the fusion is more effective in pig behavior detection, stating the significance of temporal context in interpreting animal behavior that develops over time [26]. This type of temporal modeling can be directly applied to cattle farming, where feeding, resting, walking, and social interactions are highly temporal processes. Livestock monitoring studies are also informed by methodological developments in YOLO architectures in adjacent agricultural fields. Significant improvements in precision and average precision in plant disease detection, with improved variants of YOLOv9 using adaptive sampling and improved loss functions, have verified that refinement of architecture can greatly benefit demanding visual problems. In cattle farming, adaptive spatial feature fusion that has been incorporated into YOLOv5 has demonstrated high accuracy of cattle body detection in the presence of occlusion and a complex background, and it can be used in the long-term autonomous detection of livestock in precision livestock farming systems [32]. The high-performance machine vision systems designed in intelligent pest tracking continue to provide testimony to the strength of optimized deep learning pipelines in application to real-time agricultural monitoring applications [33,34]. Taking the idea of visual object tracking to the next level, scientists integrated the YOLOv7 object detection network and Deep SORT tracking algorithm [35]. Performance analysis was carried out based on a number of criteria, and the proposed model obtained better performance indices than the YOLOv5 model. From this work, the authors establish the need for the combination of advanced object detection networks coupled with tracking algorithms for enhanced performance, especially in the case of dynamic settings. Lastly, a study for early pest detection trained six YOLO models, which include YOLOv3, YOLOv3-Tiny, YOLOv4, YOLOv4-Tiny, YOLOv6, and YOLOv8, with a 9875-sample image collected under varying illumination and annotated in the YOLO format [36,37]. Regarding the YOLOv8 model, it was executed for real-time pest detection in an Android application and it gave the best mAP of 84.7%. As has been demonstrated in this paper, YOLO models can be successfully used for pest identification to further explore the practical application in agriculture.

## 3. Proposed Methodology

In this section, we will comprehensively discuss each phase of the proposed methodology, including data collection, data preprocessin, model design, model training, and validation.

### 3.1. Data Collection

**Figure 1:**
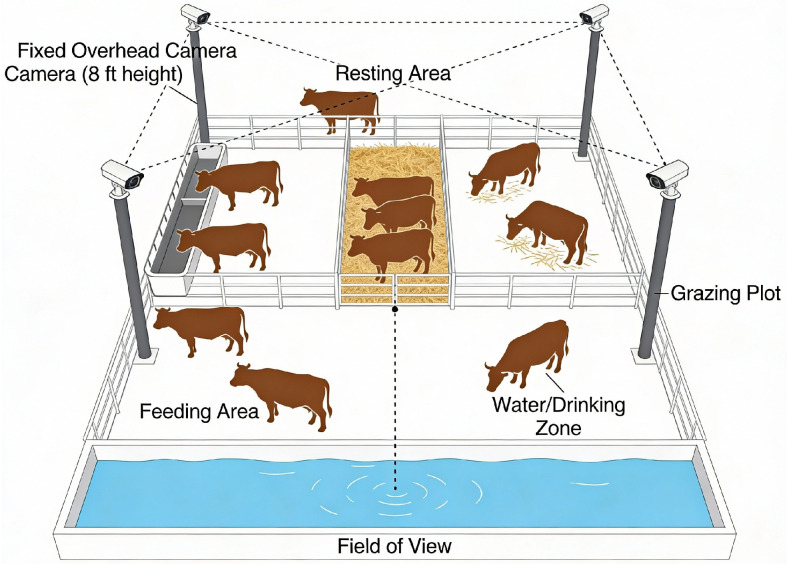
Image data collection scenario.

The initial phase of the study was devoted to creating a representative and detailed dataset of cattle images. The Nestlé cattle farm was used to gather data, with high-resolution cameras fixed on pillars, to track the dynamics of activities involving over 300 cows. The final dataset comprises 2956 images, including 2300 images acquired from the Nestlé dairy farm and 656 images obtained from online sources to broaden the variation in breeds and background scenes, while retaining the same four behavioral categories (standing, lying, grazing, and estrus). Farm images were recorded using fixed overhead cameras mounted at approximately 8 ft across multiple locations (feeding, resting, drinking, and open walking areas) and under varying times of day/night and weather conditions. This tactical use was necessary in order to capture natural behavioral patterns and interactions in the herd. A general architectural installation of the image data collection process in terms of its spatial arrangement and overall architectural setup is depicted in Figure 1.

**Figure 2:**
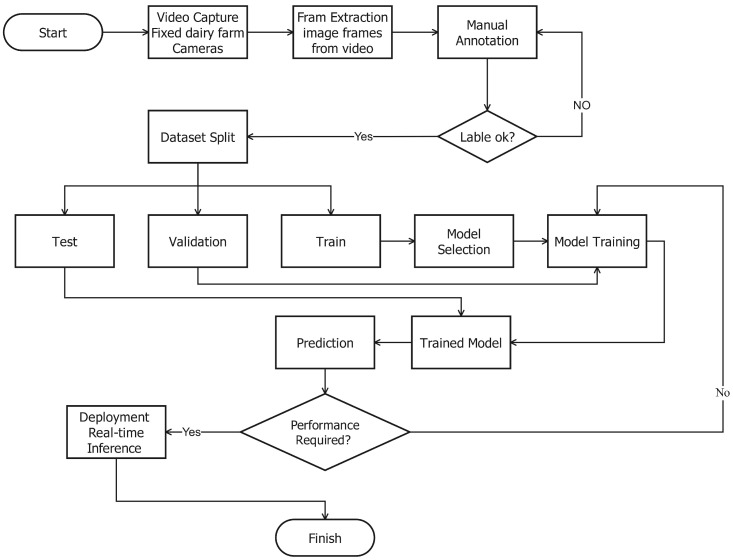
Detection workflow.

The images of the Nestlé farm were taken under various environmental conditions of the environment and at various times of the day and weather settings. The diversity of the environment, i.e., sunny, cloudy, rainy conditions, and night conditions, ensures that the trained models can be resistant and flexible to the real-life situations that involve practical deployment in the working farms. In particular, the images that were obtained via the internet depicted various types of cattle breeds, showing cattle in vast settings, such as mountainous settings, flat plain settings, and inside feeding stations, among others, which increased the environmental variety of data. One of the major aspects of the data collection plan was making sure that they included images of different behavioral states, which, in this case, were standing, sitting, grazing, and estrus. A combination of images of the Nestlé farm, taken directly and those found on the internet, makes a balanced and complete dataset. The result of models that are trained under this type of varied data is more likely to work well in other farms, other cattle breeds, and other environmentally related factors, and thus make them more applicable in large-scale cattle health monitoring, the workflow for this is shown in Figure 2.

Although the dataset contains approximately 3000 annotated frames, each image includes multiple animals with fine-grained behavioral labels. Estrus behavior, however, is a rare, short-duration, and non-periodic event, which makes it intrinsically challenging to capture under continuous farm monitoring conditions. As a result, reliable estrus annotation necessitates extended observation and confirmation by veterinary experts, which constrains the feasibility of large-scale data collection. The resulting dataset, therefore, reflects a quality-oriented, event-focused sampling design rather than maximization of sample size, providing robust supervision for this critical behavioral state.

### 3.2. Dataset Annotation

**Figure 3:**
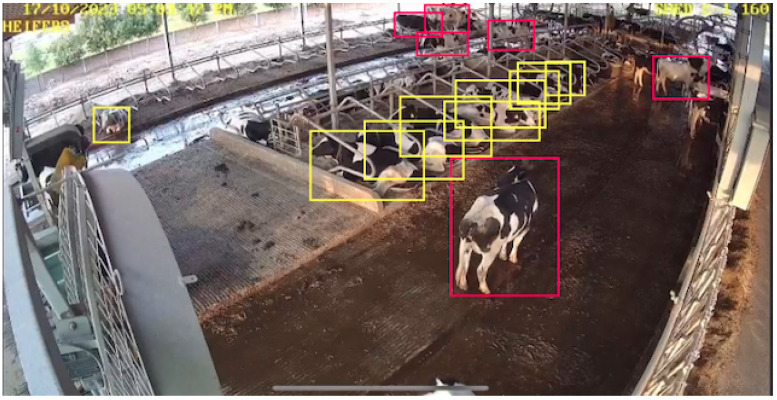
Image Roboflow annotations for cattle behavior detection, with red boxes indicating standing and yellow boxes indicating lying in a real farm environment.

The quality, diversity, and completeness of the training dataset are the basic elements of any object detection and classification task’s success. In recognition of this important need, the current work thoroughly developed and created a solid dataset that is concerned with cattle behavior observation. The variety of farm locations ensured that data would represent a wide range of real-life scenarios, thus increasing the external validity of the trained models to unresearched situations. The resulting set of video frames is 2956 in size, and every video frame was manually annotated to reflect four different cattle behavior classes that are of great importance in livestock surveillance. These annotations have been performed through Roboflow, which is a mature and popular computer vision data management system, as shown in Figure 3. Roboflow has provided fine-grained annotation, which means that fine-tuning bounding boxes for every cow in the image can tell the spatial scale and the location of every cow. The bounding boxes were manually associated with one of the existing behavior class labels, such as standing, lying, grazing, or estrus, which were listed during the annotation procedure. To achieve consistency, accuracy, and reliability, the rigorous labeling strategy was used in the entire dataset. The meticulous classification of cattle activities helps the creation of deep learning models that can help discern between minor changes in animal activity reliably, thus advancing the use of intelligent monitoring solutions addressing the agricultural domain.

**Standing:** This class features frames depicting cows that either stand at rest or reveal slight alterations in weight. Precise annotation involved special consideration to body posture and pose, which defines standing behavior.

**Lying:** Frames in this class consist of lying cows, lying down as they normally do, in their relaxed and restful pose. The emphasis of the annotation was on the exact portrayal of the entire body of the cow when resting.

**Grazing:** This class will include shots that are taken of cows actively grazing on grass or other feed. Efficient labeling of the grazer is needed to determine the posture of the characteristic head and body position related to the activity of the grazer.

**Estrus:** Frames that are under this category depict cows with behavioral signs of heat. They include unique body language patterns, augmented movement, and particular physical gestures. These behavioral cues are fine and so delicate, and they require total attention to annotate this class.

In order to achieve an extremely high rate of annotation accuracy and consistency, Roboflow was utilized during the process of labeling. The intuitive user interface ensured accurate assignment of bounding boxes and behavior labeling, as well as enabled a systematic and error-free annotation process. All the bounding boxes were checked to ensure that they matched their respective behavior class, making it high-quality data that can be used to make strong and dependable cattle behavior classification models. It is also important to note that the estrus images are manually annotated first to provide sufficient labeled frame data to the machine to learn each piece of information. This directly addresses the ambiguity of the false annotation and ensures the provision of an accurately correct dataset. To assess annotation consistency, a quantitative verification was conducted during the second-stage review process. A stratified subset of the dataset, with emphasis on the estrus class, was independently re-evaluated during expert verification. The resulting label agreement exceeded 90%, with most discrepancies occurring in visually ambiguous frames involving partial occlusion, brief mounting-like interactions, or transitional postures between standing and estrus. These cases reflect the intrinsic difficulty of frame-level estrus annotation rather than systematic labeling errors.

In Table 2, a summary of the dataset structure is given, and it shows the count of images per behavior or category. The resulting breakdown gives one an idea of the distribution of the classes, which is one of the key requirements when training effective object detection models. Such a balanced distribution can be useful in avoiding the overproduction of bias to the superiority of the dominant classes and will work to enhance the performance of the models. Also, the table provides a consistent uniform image resolution of 1280 × 800 pixels, and there was no variation in this parameter throughout the dataset. Fixed resolution reduces the amount of extra processing that might be required to detect artifacts in the form of resizing and other preprocessing activities, which might negatively impact the accuracy of the detection.

In order to increase the integrity of the datasets further, very stringent quality control guidelines were followed in the annotation process. The two-stage validation method was used. First, a top annotator coded a limited number of items on the data with a lot of care. Later, we revised and checked the annotations again. Any differences or conflicts were to be sorted out in a systematic conversation and agreement. This strict verification process considerably minimized annotation errors and ensured that the resulting set of data was of high quality, demanded to train reliable models in cattle behavior recognition and classification.

### 3.3. Dataset Split

Concretely, the dataset was split into 70% training, 15% validation, and 15% testing at the image level. All 2300 farm images were randomly partitioned into train/validation/test with non-overlapping recording sessions, while all 656 internet images were assigned only to the training and validation sets, with none included in the test set. The resulting test set, therefore, contains only Nestlé farm images and reflects the actual deployment environment.

### 3.4. YOLO Variants for Cattle Behavior Classification

This paper presents the exploration of how two popular members of the YOLO (You Only Look Once) models of object detection, i.e., YOLOv8 and YOLOv9, can be used to classify cattle behavior. It is well known that YOLO-based models are effective in object detection in real time and with high accuracy, which is why they are specifically applicable in livestock monitoring applications where prompt detection and accuracy are the most important attributes.

#### 3.4.1. YOLOv8

**Figure 4:**
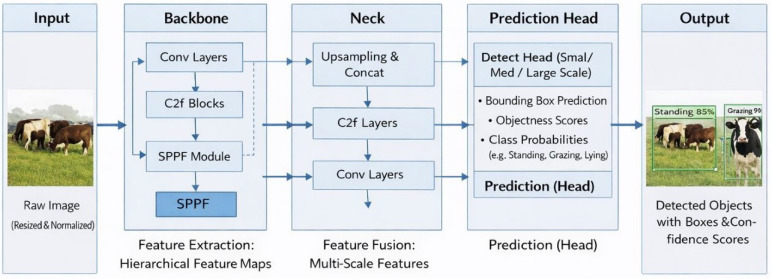
Pipeline of a YOLO-based object detection model.

YOLOv8 is a well-balanced model with trade-offs between the detection performance and the computational needs. It uses a number of architectural enhancements capable of increasing the capability of the model to extract meaningful features from the input images. These improvements incorporate the existence of sophisticated convolutional layers that are deliberately created to extract fine-grained spatial data. These layers help the model to learn the discriminative patterns effectively and distinguish between cattle and the background, in addition to differentiating the behaviors of different cattle. Moreover, YOLOv8 uses an optimized detection head, which is set to produce accurate bounding boxes on the detected instances of cattle. This detecting head uses the extracted feature representations to be able to predict the location, size, and the corresponding behavior class of each cow in a frame carefully. Figure 4 shows the general design of YOLOv8, displaying the pipeline of an object detector model that is based on the YOLO concept.

#### 3.4.2. YOLOv9

YOLOv9 includes the most recent developments on the YOLO architecture and has additional benefits both to the model efficiency and to the detection performance. YOLOv9 introduces architectural refinements designed to enhance detection accuracy and computational efficiency in complex visual scenes. Relative to YOLOv8, YOLOv9 employs improved backbone and neck architectures, combined with more effective feature aggregation mechanisms, to more reliably detect small-scale and partially occluded objects, which frequently occur in dense herd configurations. These architectural advances are further supported by revised training strategies that promote more stable optimization and improved generalization under limited-data conditions. In this study, four variants of YOLOv9 (n, s, c, e) are trained and evaluated using the same dataset partitions, data augmentation schemes, and hyperparameters as YOLOv8, allowing a controlled comparison of architectural refinement versus added complexity for the recognition of estrus-oriented cattle behavior.

This study uses both YOLOv8 and YOLOv9 to provide a comparison of their performance in the classification of cattle behavior. Not only is the detection accuracy evaluated, but a trade-off between the speed and performance of each version of the model is also analyzed. The overall evaluation can give much information about the appropriateness of the YOLO-based models to be applied to real-world systems related to cattle monitoring, especially in analyzing the applications that need to be performed in a real-time mode and have limited computational capabilities.

### 3.5. Model Training

As the meticulously annotated data is constructed and the YOLOv8 and YOLOv9 models are selected, the next important stage of the current research is dedicated to model training and evaluation. This step is necessary since it has been demonstrated that the chosen deep learning models can successfully learn discriminative features and be properly extrapolated to previously unseen cattle behavior data.

### 3.6. Model Training: Fine-Tuning for Optimal Performance

The model training procedure consists of applying the annotated data to the chosen YOLO models and testing their parameters to obtain precise results in identifying the behavior of cattle. These are the main stages that are included in this process.

**Data Preprocessing:** Before training, several preprocessing operations are applied to the cattle dataset to enhance the performance of the YOLOv8 and YOLOv9 models. Image normalization is performed so that pixel intensity values are rescaled. This helps stabilize and speed up the optimization process during training. In addition, data augmentation techniques are used to improve generalization and reduce overfitting to specific viewpoints or lighting conditions in the cattle environment.

**Model Training:** The image set is referred to as the training set and is used to update the model parameters, and the validation set is used to track the performance throughout the training process and help alleviate overfitting. The testing set is not visible in the training process and is used in the final performance evaluation. In the training process, the YOLO models are trained to identify the bounding boxes and class labels of the behavior of cattle in every frame.

**Loss Function and Optimization:** In every training cycle, the model estimates the position of objects and the classes of their behaviors that are compared with the ground truth annotations. To measure the difference between predicted and actual bounding boxes and class labels, a loss function is employed, e.g., the Intersection-over-Union (IoU)-based loss. An optimization algorithm is used to reduce this loss, which is an Adam optimizer, which in turn periodically changes the network weights to minimize the errors in its predictions and enhances its convergence.

## 4. Model Evaluation

The model performance evaluation is, as explained above, based on well-defined metrics, such as mAP, precision, recall, and inference speed. This study can maximize the scientific rigor and credibility of the research because it explicitly specifies the experimental design and methods used in assessing the outcomes of the experimental study, and it will be reproducible by other scientists, which will further improve the scientific rigor and credibility of the research.

**Accuracy:** Accuracy is reported as the proportion of correctly classified cattle behavior instances across all evaluated frames in the farm-only test set.

(1) $$Accuracy=\frac{TP+TN}{TP+FP+TN+FN}$$

**Precision:** Precision indicates how reliably the model’s predicted cattle behavior labels, particularly estrus detections, correspond to correct instances in the farm-only test set.

(2) $$Precision=\frac{TP}{FP+TP}$$

**Recall:** Recall reflects the model’s ability to correctly identify instances of each cattle behavior, particularly the detection of estrus events.

(3) $$Recall=\frac{TP}{FN+TP}$$

**F1 Score:** The F1 score summarizes the balance between precision and recall, providing a single indicator of detection quality for cattle behavior recognition.

(4) $$F1Score=\frac{2\times Precision\times Recall}{Precision+Recall}$$

**Mean Average Precision (mAP):** mAP is used as the primary evaluation metric to summarize detection and classification performance across all cattle behavior categories, reflecting the overall precision–recall trade-off of the model.

**Inference Speed:** The speed of detection is a key factor when it comes to means of deploying it on practical levels at the farm. As a result, both YOLOv8 and YOLOv9 models are tested in terms of their inference pace on a representative platform. This analysis consists of the time that was taken by each model to run an input image and provide detection results. The speed of inference is an important factor that needs to be assessed to decide the practicality of implementing these models in real-time monitoring systems and resource-constrained environments.

### Experimental Setup

In order to have a rigorous, transparent, and reproducible assessment, a clearly defined experiment setup is used. In this section, the major parameters and settings for training and evaluation are presented in Table 3.

**Hardware and Software:** The hardware platform on which training and evaluation are conducted is clearly stated, such as the central processing unit (CPU) and graphics processing unit (GPU), and the memory available. Moreover, the specific versions of the deep learning implementation (e.g., PyTorch or TensorFlow) and the YOLO implementations (YOLOv8 and YOLOv9) used in the experiments are recorded to provide reproducibility.

**Training Parameters:** The hyperparameters used in the training of the models are clearly specified because they affect the performance of the models significantly. The egocentric hyperparameters are as follows.

## 5. Evaluation Results

The trained YOLOv8 and YOLOv9 models are tested on the unseen testing dataset after the training phase. This comparison presents an objective comparison of how the models can identify and classify cattle behaviors in real-world situations. The effectiveness of the two models is compared using performance metrics that include precision, recall, F1 score, and mean Average Precision (mAP) as well as to examine the trade-off between detection accuracy and computation efficiency.

### 5.1. Training Results

**Figure 5:**
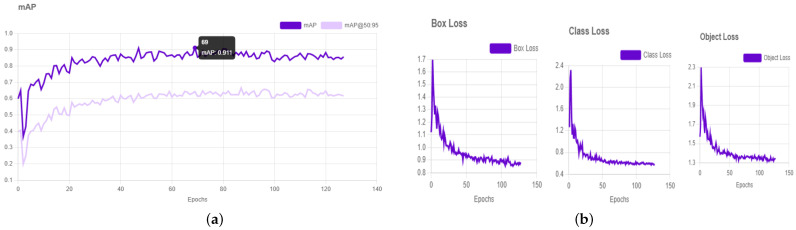
Performance (**a**) YOLOv8-L with pretrained checkpoint achieves mAP of 0.911. (**b**) Loss curves (box loss, class loss, and object loss) for a single image.

Figure 5a demonstrates the training loss behavior of the single-image input with the plot of evolution of box loss, class loss, and object loss with the epochs of training. Box loss is the precision of bounding box localization prediction, class loss is the precision of classification, and object loss is the precision of object presence prediction. The progressive convergence and progressive realization of performance improvement in the course of training are evidenced by the smooth and downward trends of these curves. In Figure 5b, the training dynamics of the YOLOv8 model are represented by three major loss goals, i.e., box loss, class loss, and object loss. Box loss is used to estimate how well the bounding boxes are predicted, class loss is used to estimate how well the behavior classification is correct, and object loss is used to estimate this feature. The successive reduction of these loss curves with increasing training epochs points to the fixed convergence and continuous enhancement of the training process, which proves the efficiency of the latter. The training and evaluation results of the YOLOv8-L model with a pretrained checkpoint demonstrate a notable performance, achieving a mean Average Precision (mAP) of 0.911% at an epoch size of 140.

### 5.2. Model Testing

YOLOv8 is offered in a variety of options, s (small), n (nano), m (medium), and l (large), as outlined in Table 4. The major distinctions between these variants include their model size, complexity of architecture, and computation requirements. A trade-off seen as a result is that there is a trade-off between the accuracy of detection and real-time performance. YOLOv8 has a balanced feature of both accuracy and speed in the case of cattle activity monitoring, in both scenarios, where intervening in the situation and managing the farm timely is essential. The balance created makes YOLOv8 especially applicable to the real-time livestock tracking application.

The YOLOv9 model also brings the object detection performance to a new level, adding Programmable Gradient Information (PGI) and Reversible Functions, which can be used to maintain the information flow between network layers. Such architectural improvements allow YOLOv9 to be highly accurate and efficient in detection, even in the case of a comparatively small dataset being trained on. Consequently, YOLOv9 proves to be acceptable in delivering real-time monitoring and works on small resources of computation, making it feasible to implement it, i e real-life farm settings. YOLOv9 is offered in a few versions, namely S (Small), M (Medium), C (Compact), and E (Extended), as indicated in Table 5. All the variants are aimed at providing a balance between accuracy, speed of inference, and cost. The S variant is lightweight and fits well in real-time execution on resource-constrained hardware, and the M and C variants are better with a moderate level of complexity in terms of detection accuracy. The E-type offers the largest representational ability and offers better detection ability at a higher computation cost.

One of the best-performing models reviewed is the YOLOv8-L model with a pretrained checkpoint, which had a final Average Precision (AP) of 91.11% when trained with an epoch size of 140. This finding illustrates how well and sound the model is in the detection and categorization of cattle behaviors.

Table 6 summarizes the relative difficulty and typical error patterns for each behavior based on detailed visual inspection of detections on the farm-only test set and the class-wise detection probabilities reported earlier (standing 90%, lying 83%, grazing 93%, estrus 83%). Due to limitations of the current evaluation tooling, exact per-class AP values cannot be exported from the training logs, so this table is presented as a qualitative, class-wise interpretation rather than a strict numerical AP breakdown.

**Figure 6:**
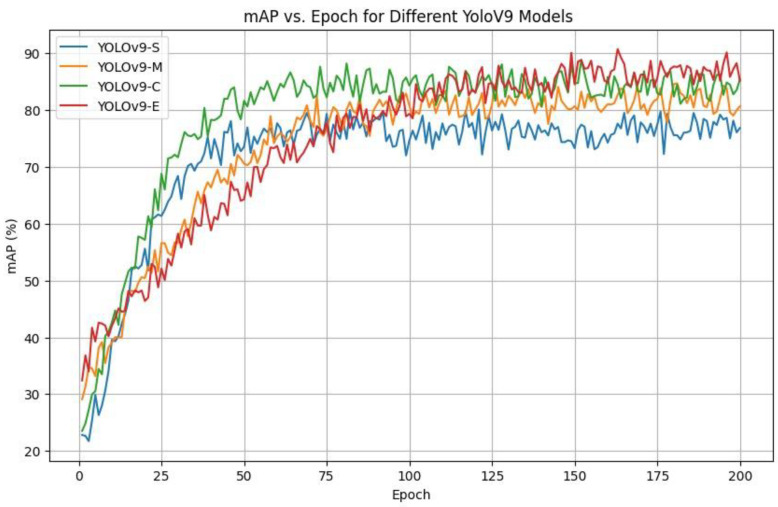
mAP vs. Epoch for different YOLOv9 models.

**Figure 7:**
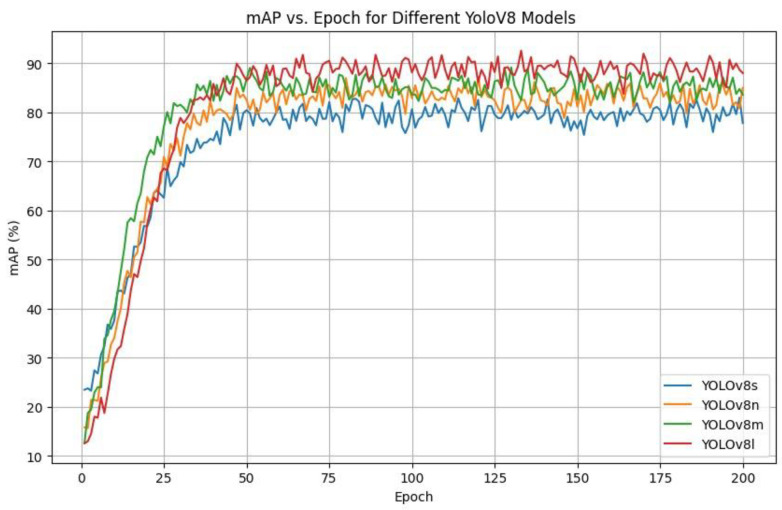
mAP vs. Epoch for different YOLOv8 models.

As a quantitative alternative, class-wise detection probabilities and precision recall trends were analyzed on the farm-only test set. These results indicate that estrus remains the most challenging category (≈83% detection probability), compared to standing (≈90%) and grazing (≈93%), which is consistent with the qualitative failure modes observed under occlusion and behavioral ambiguity. In a comparison and contrast analysis of the YOLOv9 variants of Small (S), Medium (M), and Large (L) trained in 200 epochs, one can clearly see that the YOLOv9-E model outperforms that of Small (S) and Medium (M) due to the relationship between mean Average Precision (mAP) y-axis and training epochs x-axis. Figure 6 shows that YOLOv9-E is able to attain higher mAPs than their smaller counterparts during the training period. In addition, it shows quicker and more consistent convergence, as well as superior learning effectiveness and performance among epochs. As illustrated in Figure 7, YOLOv8-l attains the highest final mAP among the YOLOv8 variants evaluated on the proposed dataset. However, its advantage over YOLOv8-m is marginal, while incurring higher inference latency, indicating diminishing returns from further increasing model capacity on this modest-scale, noise-prone livestock dataset. These findings indicate that medium-sized YOLOv8 models already offer a favorable compromise between detection accuracy and real-time efficiency for barn deployment, rather than larger capacity alone yielding substantial performance gains. Within the YOLOv9 family, YOLOv9-E achieves the highest mAP among the examined small, medium, and compact configurations. However, the improvement over YOLOv9-C remains moderate, and the extra-large variant introduces a pronounced increase in computational cost, thereby limiting the practical benefit of additional capacity in this context. This controlled comparison suggests that architectural scale constitutes only one determinant of performance, and that compact or medium-capacity YOLOv9 models may be more suitable when stringent latency constraints prevail in commercial farm environments. Across both YOLOv8 and YOLOv9, performance does not increase monotonically with model size: medium and compact variants already attain competitive mAP and class-wise precision and recall, whereas the largest models provide only incremental gains at distinctly higher inference times. Under identical training and evaluation protocols, these results demonstrate that increased capacity alone is insufficient to guarantee superior fine-grained behavior recognition on this dataset, and that model selection must explicitly account for the accuracy–latency trade-off and deployment constraints.

The higher performance of YOLOv8-L is mainly because of its more complex structure, which allows the model to extract richer and more discriminative features out of the data. Even though this added complexity requires more computational tools, it also translates into a much higher level of accuracy and detection reliability. These results highlight the benefit of using bigger YOLO variants in a problem where accuracy and resilience are important. All quantitative results reported in this section are obtained on the farm-only test set, which contains 15% of the Nestlé farm images and excludes all internet-sourced images, in order to reflect realistic deployment conditions and avoid domain leakage from web imagery.

### 5.3. Inference on Videos and Images

To evaluate real-time suitability, inference latency was quantified for the YOLOv8 and YOLOv9 variants on a workstation equipped with an Intel^®^ Core™ i9-14900KF 3.20 GHz, an NVIDIA GeForce RTX 5070, and 32 GB of system memory. All benchmarks were conducted with a batch size of 1 at an input resolution of 1280×800 pixels, corresponding to the native frame size of the Nestlé farm cameras, and encompassed the full pipeline from image loading and resizing, through the forward pass, to confidence thresholding and non-maximum suppression (NMS). Under these conditions, YOLOv8-L achieves a mean end-to-end latency of 50 ms per frame (≈20 FPS), whereas YOLOv9-E exhibited 69 ms per frame (≈14.5 FPS); smaller configurations (e.g., YOLOv8-s) achieved higher frame rates at the expense of a modest reduction in accuracy. These processing rates meet typical demands for continuous barn monitoring (5–10 FPS) and provide sufficient margin for additional application-side computation in a farm deployment setting. The behavioral analysis was based on inference conducted on four classes of standing, estrus, lying, and grazing. The model detected standing 90% of the time, estrus 83% of the time, lying 83% of the time, and grazing 93% of the time, indicating credible bounding box localization and classification capability on all the target behaviors. These findings confirm the efficiency of the YOLO-based models used in real-time cattle activity tracking in the feasible farm settings. In the case of the standing class, the YOLOv8-L model had a detection probability of 90% in detecting and marking standing cattle in various scenes. It is a high confidence that indicates the strength of the model to identify upright positions in both different environmental conditions and, therefore, its reliability in minimizing the model in real-life applications in farms. Moreover, the length of time to infer standing behavior was steady, which makes the model computationally effective. In the case of the estrus class, the model attained a detection probability of 83% for accurate bounding box predictions. Although this value is slightly lower than that observed for the standing class, it still represents a strong performance, enabling reliable identification of estrus-related behaviors. The inference time remained stable across evaluations, further emphasizing the suitability of the model for real-time estrus monitoring and behavioral analysis. The same lying class had a detection probability of 83%, which is comparable to that of estru behavior. This stability within several categories of the behavior is evidence of the balanced detection of the YOLOv8-L model. It was also found to have stable inference times in this class, and this is proof that it is possible to roll out the model in real-time livestock tracking systems.

**Figure 8:**
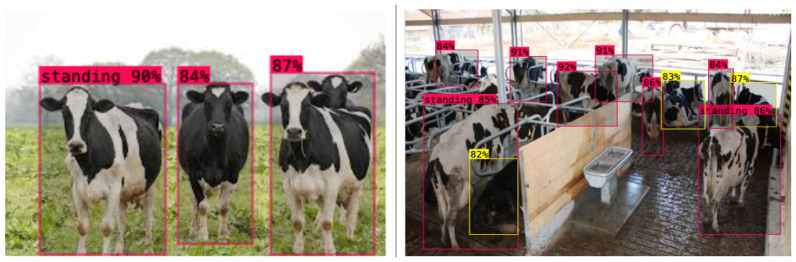
Lying and Standing scenario class—91% probability.

**Figure 9:**
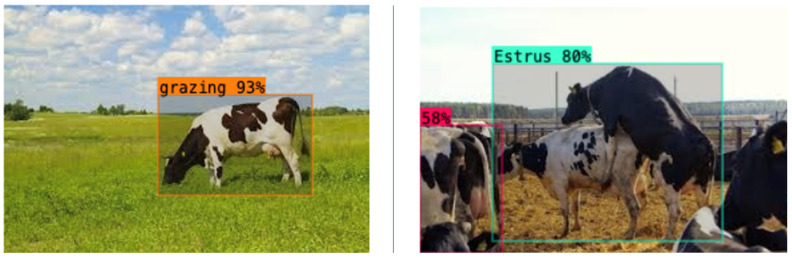
Estrus and Grazing scenario class—80% probability.

The greatest accuracy of detection was found with regard to the grazing type, with a likelihood of 93%. This is an outstanding outcome that highlights the accuracy of the model in determining the grazing behavior, which is the most critical indicator of cattle health and well-being. The overall system efficiency was supported as inference times were the same as with the rest of the classes. To further determine the strength and external validity of the YOLOv8-L model, more testing was conducted on randomly chosen images obtained on the internet, which are not part of the controlled experimental data. The model has yielded high accuracy and reliable performance in all of the assessed scenarios, such as standing, estrus, lying, and grazing (Figure 8 and Figure 9). These findings support the fact that the model can be generalized to different and unstructured environments. The overall stability inference time with high detection rates in all classes also confirms the effectiveness, reliability, and practical abilities of the YOLOv8-L model to be a reliable, efficient, and realistic method of cattle behavior monitoring and object detection tasks.

## 6. Results Discussion

In the proposed estrus-oriented cattle behavior dataset comprising 2956 images, YOLOv8-L achieves the highest mean Average Precision (mAP) of 0.9115, marginally outperforming YOLOv9-E (0.9032), despite YOLOv9 representing a more recent architecture. This slight performance advantage can be attributed to the interaction among model complexity, dataset size, and behavioral label characteristics. Specifically, with only 406 estrus images, frequent occlusions, and heterogeneous barn environments, the higher-capacity YOLOv9-E is more prone to overfitting subtle annotation noise and farm-specific visual patterns. In contrast, the more mature YOLOv8-L backbone provides a more favorable bias–variance trade-off under limited data conditions. Class-wise analysis further indicates that grazing is the easiest behavior to detect (approximately 93% detection probability), owing to its distinctive head-down posture and relatively stable feeding context. Conversely, estrus is the most challenging class (approximately 83%), primarily due to pronounced class imbalance, subtle and intrinsically temporal visual cues, and recurrent occlusions that lead to confusion with standing or playful mounting behaviors. For routine monitoring of standing, lying, and grazing, the approximately 0.9 percentage-point difference in mAP between YOLOv8-L and YOLOv9-E is unlikely to affect daily farm management decisions, as both models deliver high accuracy and real-time inference capability (approximately 20 FPS for YOLOv8-L versus approximately 14.5 FPS for YOLOv9-E at an input resolution of 1280×800). In contrast, for estrus detection, the combination of slightly higher accuracy and lower inference latency renders YOLOv8-L the more suitable deployment choice, as reducing missed estrus events has direct economic consequences. In contrast to the studies summarized in Table 1, which frequently report performance gains from newer YOLO variants trained on larger or synthetically augmented datasets, these results indicate that, for fine-grained estrus-focused cattle monitoring under realistic farm conditions, data quality and task alignment may have a greater influence on performance. This observation provides a practical implication for behavior-specific livestock datasets, architectural refinement, and judicious deployment design that may exert a greater influence on performance than enlarging model capacity in isolation. Consequently, the most recent detector does not necessarily yield the best in-field performance.

## 7. Limitations and Deployment Considerations

Although the proposed system demonstrates robust performance on the Nestlé dairy farm dataset, several limitations must be considered when assessing its applicability beyond the studied environment. The model was trained and evaluated primarily using data from a single commercial farm; consequently, its generalization to farms with differing barn architectures, cattle breeds, camera elevations, and background configurations has not yet been empirically established. Substantial variations in camera placement or environmental structure may therefore require domain-specific adaptation. The system is also sensitive to illumination and weather variability, particularly in scenarios involving strong backlighting, low-light conditions, or rain-induced motion blur. While the dataset includes imagery captured under a range of environmental conditions, the current evaluation protocol relies on a farm-specific test set that does not explicitly stratify performance across daytime and nighttime or favorable and adverse weather conditions. In addition, high animal density and frequent occlusions in congested feeding or resting areas continue to challenge detection accuracy, leading to missed instances and occasional ambiguity between visually similar behaviors. This effect is most pronounced for lying and estrus, where only partial body postures may be observable. Moreover, estrus represents an inherently temporal and physiological process, whereas the present framework infers estrus from individual frames without explicit temporal modeling or integration of physiological or auxiliary sensing information. Residual annotation uncertainty may persist, particularly for the estrus category, which is visually subtle and evolves over time. Such label ambiguity may partially explain the comparatively lower recall observed for estrus relative to other behaviors. This issue will be addressed in future work through multi-expert annotation strategies and the adoption of formal inter-annotator agreement measures. A further limitation of the current evaluation is the absence of per-class Average Precision (AP) metrics for individual behavior categories. Although overall mean Average Precision (mAP) and class-wise precision and recall provide meaningful indicators of system performance, the lack of per-class AP restricts fine-grained quantitative analysis, especially for estrus. Future studies will incorporate tailored evaluation pipelines to enable per-class AP reporting and sequence-level metrics, which are essential for accurately characterizing temporally evolving behaviors. Finally, as all quantitative results are derived from data collected at a single commercial farm, the reported findings should be interpreted within this specific operational context.

## 8. Conclusions

The paper has made a thorough comparative analysis of deep learning-based object detectors, including YOLOv8 and YOLOv9, to monitor cattle activities on camera-based farms. By its experimental outcomes, both of the models are highly accurate and profitable in key cattle behavior recognition, which proves their capabilities and appropriateness in terms of real-time observation on the farm and automated analysis of animal activities. YOLOv9, with its superior architectural design and optimization methods, showed better detection rates and higher performance stability, especially under complex scenes and detecting small objects, as this method was among the tested methods. The results highlight the increasing role of artificial intelligence in contemporary livestock production, where manual inspection regimes are progressively complemented and supplemented by intelligent, vision-based scans. Deep learning-based methods can help to enhance the welfare of animals, to confirm the early rate of detecting abnormalities related to health, and to make more informed decisions in farm management since they can monitor the behavior of cattle, scale up, and perform all according to the circumstances without imposing any devices on the animals. The results of this study demonstrate the effectiveness of YOLO-based detection models for estrus-oriented cattle behavior recognition within the studied commercial farm environment. While the proposed dataset and evaluation protocol provide a realistic test bed for deployment-oriented analysis, quantitative assessment of cross-farm generalization is beyond the scope of the present work and remains an important direction for future research. Overall, this work should be interpreted as a deployment-oriented evaluation study that bridges the gap between generic object detection benchmarks and real-world precision livestock farming requirements, rather than as a proposal of a new detection architecture.

## 9. Future Work

In view of these limitations, including class-specific performance differences in which estrus remains the most challenging behavior due to visual ambiguity and temporal evolution, subsequent work will proceed along four complementary directions that directly address the identified constraints. First, the dataset will be extended to encompass multiple farms, breeds, and barn geometries, enabling systematic cross-farm evaluation and domain-adaptation studies to quantify and enhance generalization beyond the Nestlé deployment site. Second, the current frame-based detector will be augmented with temporal and multimodal fusion, integrating YOLO-based visual signals with accelerometers, GPS, RFID, and environmental sensors so that estrus and other complex behaviors can be inferred from joint appearance, motion, and physiological context, as advocated in recent livestock monitoring literature. Third, additional camera configurations, including multi-view and combined overhead-plus-lateral setups, will be investigated to alleviate occlusion and crowding in feeding and resting areas, thereby improving class separability for lying and estrus in dense herds. Finally, a systematic benchmark will be undertaken against newer detector families (e.g., YOLOv10–YOLOv12, transformer-based models such as RF-DETR and refined RT-DETR variants, and later edge-oriented architectures like YOLO26), together with pruning, quantization, and lightweight designs, to identify architectures that offer an optimal accuracy–latency compromise on realistic farm-grade hardware and to establish a clear state-of-the-art for fine-grained cattle behavior monitoring.

## Figures and Tables

**Table 1:** Comparison of related work in deep learning-based livestock monitoring.

Author (Year) (Reference)	Species	Live	Dataset	Task Objective	Method/Model	Size	RT	YOLOv9	mAP
Bumbálek et al. (2025) [21]	Chicken	**✗**	Synthetic	Dead chicken detection	YOLOv8, YOLOv9, YOLOv10, YOLOv11	Small	**✓**	**✓**	0.983
Čakić et al. (2025) [22]	Chicken	**✗**	Hybrid	Chicken detection	YOLOv9	Small	**✓**	**✓**	0.829
Lu & Wang (2024) [23]	Plants	**✗**	Public	Multi-crop detection and counting	MAR-YOLOv9	Small	**✓**	**✓**	0.899
Shin et al. (2025) [24]	Cattle	**✓**	Custom	Lesion and inactivity detection	RT-DETR, YOLOv8	–	**✓**	**✗**	0.835
Ji-Hyeon Lee et al. (2024) [25]	Pig	**✓**	Custom	Sow and piglet behavior detection	YOLOv7, YOLOv9 with ELAN	–	**✓**	**✓**	0.796
Qin et al. (2025) [26]	Pig	**✓**	Custom	Pig behavior recognition	Enhanced YOLOv5	–	**✓**	**✗**	0.81
Andika et al. (2025) [27]	Pig	**✓**	Custom	Pig feces detection	YOLOv9	–	**✓**	**✓**	0.705
Peng et al. (2024) [14]	Yak	**✓**	Custom	Live body weight estimation	YOLOv8	–	**✓**	**✗**	0.990
Pei et al. (2024) [15]	Cattle/Sheep	**✓**	Custom	Cattle and sheep target detection	YOLOv8-ZX, YOLOv9	–	**✓**	**✓**	0.89
Das et al. (2024) [17]	Cow	**✓**	Public (COLO)	Indoor cow localization	YOLOv8, YOLOv9	S/L	**✓**	**✓**	0.70
Zhang et al. (2025) [28]	Sheep	**✓**	Custom	Facial recognition and health monitoring	Modified YOLOv5s	Small	**✓**	**✗**	0.958
**Proposed Work**	**Cattle**	**✓**	**Custom (Nestlé Farm)**	**Activity monitoring: standing, lying, grazing, estrus**	**YOLOv8 and YOLOv9 (N/M/L variants)**	**S–L**	**✓**	**✓**	**91.11%**

**✓** = Yes, **✗** = NO.

**Table 2:** Dataset composition.

Class	Number of Images	Size (Pixels)
Standing	950	1280 × 800
Lying	800	1280 × 800
Grazing	800	1280 × 800
Estrus	406	1280 × 800

**Table 3:** Experimental Settings.

Parameter	YOLOv8	YOLOv9
Annotation format	XML	XML
Image size (pixels)	1280×800	1280×800
Batch size	16	16
Epochs	200	200
Learning rate	0.01	0.01
Optimizer	Auto	Auto
Confidence threshold	0.25	0.25
CPU	Intel® Core™ i9-14900KF @ 3.20 GHz	
System RAM	32 GB	
GPU	NVIDIA GeForce RTX 5070	
CUDA Version	CUDA 11.7+	
cuDNN	cuDNN 8.x	
Deep Learning Framework	PyTorch 1.13+	
Augmentations	Crop: 0% min zoom, 20% max zoom	
	Rotation: $-15^{\circ }$ to $+15^{\circ }$	
	Saturation: −25% to +25%	
	Exposure: −10% to +10%	

**Table 4:** Results of YOLOv8 variants: model performance comparison.

Model	Input Size	Precision	Recall	F1 Score	mAP
YOLOv8-s	800×800	0.7872	0.8463	0.8720	0.8523
YOLOv8-n	800×800	0.8276	0.8563	0.8776	0.8612
YOLOv8-m	800×800	0.8812	0.8732	0.8642	0.8745
YOLOv8-l	800×800	0.9260	0.8875	0.9010	0.9115

**Table 5:** Results of YOLOv9 variants: model performance comparison.

Model	Input Size	Precision	Recall	F1 Score	mAP
YOLOv9-s	800 × 800	0.7934	0.8783	0.8820	0.8723
YOLOv9-m	800 × 800	0.8935	0.8828	0.8966	0.8830
YOLOv9-c	800 × 800	0.9033	0.8972	0.8993	0.8928
YOLOv9-e	800 × 800	0.9128	0.9075	0.8949	0.9032

**Table 6:** Class-wise detection tendencies of the YOLOv8-L model on the farm-only test set, combining quantitative detection rates with qualitative error analysis.

Class	Relative Difficulty	Typical Errors	Notes
Standing	Easy	Confusion with estrus in restless cows	High precision/recall globally
Lying	Medium	Missed under heavy occlusion	Affected by crowded barns
Grazing	Easy	Rare confusion with standing	Highest detection reliability
Estrus	Hardest	Missed when partially occluded; false positives in playful mounting	Most clinically critical

## Data Availability

The original data presented in the study are openly available in Kaggle at https://www.kaggle.com/datasets/rajaasim7252/cattle-activity-monitoring accessed on 23 January 2026.

## Abbreviations

The following abbreviations are used in this manuscript:

AI	Artificial Intelligence
ML	Machine Learning
DL	Deep Learning
CNN	Convolutional Neural Network
YOLO	You Only Look Once
IoT	Internet of Things
CV	Computer Vision
PLF	Precision Livestock Farming
RGB	Red–Green–Blue
UAV	Unmanned Aerial Vehicle
GPS	Global Positioning System
RT	Real Time
FPS	Frames Per Second
mAP	Mean Average Precision
IoU	Intersection over Union
TP	True Positive
FP	False Positive
TN	True Negative
FN	False Negative
SPPF	Spatial Pyramid Pooling Fast
C2f	Cross-Stage Partial Fusion
YOLOv8	Eighth version of the YOLO object detection architecture
YOLOv9	Ninth version of the YOLO object detection architecture
YOLOv8-S	YOLOv8 Small variant
YOLOv8-M	YOLOv8 Medium variant
YOLOv8-L	YOLOv8 Large variant
YOLOv9-S	YOLOv9 Small variant
YOLOv9-M	YOLOv9 Medium variant
YOLOv9-E	YOLOv9 Enhanced variant
RT-DETR	Real-Time Detection Transformer
ELAN	Efficient Layer Aggregation Network
